# The ST2^+^ Treg/amphiregulin axis protects from immune-mediated hepatitis

**DOI:** 10.3389/fimmu.2024.1351405

**Published:** 2024-03-20

**Authors:** Selina Wachtendorf, Fitriasari Jonin, Aaron Ochel, Fabian Heinrich, Astrid M. Westendorf, Gisa Tiegs, Katrin Neumann

**Affiliations:** ^1^ Institute of Experimental Immunology and Hepatology, University Medical Center Hamburg-Eppendorf, Hamburg, Germany; ^2^ Hamburg Center for Translational Immunology, University Medical Center Hamburg-Eppendorf, Hamburg, Germany; ^3^ Institute of Medical Microbiology, University Hospital Essen, University of Duisburg-Essen, Essen, Germany

**Keywords:** Treg maintenance and function, ST2^+^ Tregs, ILC2s, amphiregulin, hepatic immunoregulation, liver inflammation

## Abstract

**Introduction:**

The alarmin IL-33 has been implicated in the pathology of immune-mediated liver diseases. IL-33 activates regulatory T cells (Tregs) and type 2 innate lymphoid cells (ILC2s) expressing the IL-33 receptor ST2. We have previously shown that endogenous IL-33/ST2 signaling activates ILC2s that aggravate liver injury in murine immune-mediated hepatitis. However, treatment of mice with exogenous IL-33 before induction of hepatitis ameliorated disease severity. Since IL-33 induces expression of amphiregulin (AREG) crucial for Treg function, we investigated the immunoregulatory role of the ST2^+^ Treg/AREG axis in immune-mediated hepatitis.

**Methods:**

C57BL/6, ST2-deficient (Il1rl1^-/-^) and Areg^-/-^ mice received concanavalin A to induce immune-mediated hepatitis. Foxp3Cre^+^ x ST2fl/fl mice were pre-treated with IL-33 before induction of immune-mediated hepatitis. Treg function was assessed by adoptive transfer experiments and suppression assays. The effects of AREG and IL-33 on ST2^+^ Tregs and ILC2s were investigated *in vitro*. Immune cell phenotype was analyzed by flow cytometry.

**Results and discussion:**

We identified IL-33-responsive ST2^+^ Tregs as an effector Treg subset in the murine liver, which was highly activated in immune-mediated hepatitis. Lack of endogenous IL-33 signaling in Il1rl1^-/-^ mice aggravated disease pathology. This was associated with reduced Treg activation. Adoptive transfer of exogenous IL-33-activated ST2^+^ Tregs before induction of hepatitis suppressed inflammatory T-cell responses and ameliorated disease pathology. We further showed increased expression of AREG by hepatic ST2^+^ Tregs and ILC2s in immune-mediated hepatitis. Areg^-/-^ mice developed more severe liver injury, which was associated with enhanced ILC2 activation and less ST2^+^ Tregs in the inflamed liver. Exogenous AREG suppressed ILC2 cytokine expression and enhanced ST2^+^ Treg activation *in vitro*. In addition, Tregs from Areg^-/-^ mice were impaired in their capacity to suppress CD4^+^ T-cell activation *in vitro*. Moreover, application of exogenous IL-33 before disease induction did not protect Foxp3Cre^+^ x ST2fl/fl mice lacking ST2^+^ Tregs from immune-mediated hepatitis. In summary, we describe an immunoregulatory role of the ST2^+^ Treg/AREG axis in immune-mediated hepatitis, in which AREG suppresses the activation of hepatic ILC2s while maintaining ST2^+^ Tregs and reinforcing their immunosuppressive capacity in liver inflammation.

## Introduction

Immune-mediated liver diseases such as autoimmune hepatitis (AIH) are characterized by hepatic inflammation, tissue destruction and development of fibrosis. The relevance of immunological pathways in disease pathology has been highlighted by genome-wide association studies, identifying risk loci that host genes involved in innate and adaptive immunity (1). However, immune-mediated processes leading to liver inflammation and immunoregulation are still poorly defined.

IL-33 functions as an alarmin, which is released as a local danger signal to alert the immune system (2). IL-33 is the only ligand of the suppression of tumorigenicity (ST)2 receptor (3), and activates ST2-expressing immune cells such as type 2 innate lymphoid cells (ILC2s) and regulatory T cells (Tregs) (4). IL-33-activated ILC2s express type 2 cytokines (5) and the epidermal growth factor amphiregulin (AREG), that contributes to tissue regeneration (6–8). IL-33 further induces activation of ST2^+^ Tregs, an effector Treg subset (9) that predominantly localizes in non-lymphoid tissue (10). IL-33 stimulates expression of AREG in Tregs and both, IL-33 and AREG ensure maintenance of Treg function during inflammation (11, 12). Whether ST2^+^ Tregs and their effector molecule AREG are important for immunoregulation in immune-mediated hepatitis is not known so far.

Elevated IL-33 levels have been described in patients with acute and chronic liver disease (13), including AIH (14), suggesting that IL-33 contributes to liver disease pathology. In the murine model of concanavalin (Con)A-induced immune-mediated hepatitis, which shares some similarities with acute AIH (15), hepatocytes have been shown to increase expression of IL-33 (16). We have recently demonstrated that formation of necrotic lesions in immune-mediated hepatitis was associated with the release of IL-33 in liver tissue and an aggravation of the disease through activation of hepatic ILC2s (17). Moreover, blockage of endogenous IL-33 was found to ameliorate liver injury (18), further supporting the pro-inflammatory role of IL-33 in immune-mediated hepatitis. In contrast, IL-33- and ST2-deficient mice developed more severe liver injury (19, 20), pointing to a protective effect of endogenous IL-33/ST2 in immune-mediated hepatitis. We have further shown that targeting the IL-33/ST2 axis by application of exogenous IL-33 suppressed immune-mediated hepatitis, which correlated with an expansion of ST2^+^ Tregs in the liver (17).

In this study, we investigated the role of the ST2^+^ Treg/AREG axis in the pathogenicity of immune-mediated hepatitis. IL-33-responsive ST2^+^ Tregs constituted a hepatic effector Treg subset that suppressed liver inflammation upon activation with exogenous IL-33. Hepatic ILC2s and ST2^+^ Tregs expressed AREG in immune-mediated hepatitis. *Areg* ^-/-^ mice developed more severe liver injury, that was associated with increased ILC2 activation and less ST2^+^ Tregs in the inflamed liver. Treatment of mice with exogenous IL-33 elevated AREG expression in ILC2s and ST2^+^ Tregs and led to reduced effector T-cell activation in immune-mediated hepatitis. The immunosuppressive effect of exogenous IL-33 was abrogated in mice lacking ST2^+^ Tregs. Thus, the ST2^+^ Treg/AREG axis regulates inflammatory immune responses in immune-mediated hepatitis.

## Materials and methods

### Mice

*Areg* ^-/-^ mice (Areg^tm1Dle^/J) were provided by Matias A. Avila (Hepatology Program, CIMA, Universidad de Navarra, Pamplona, Spain). *Il1rl1* ^-/-^ mice (Il1rl1^tm1Anjm^) were provided by Max Löhning (Experimental Immunology and Osteoarthritis Research, Department of Rheumatology and Clinical Immunology, Charité-Universitätsmedizin Berlin, Berlin, Germany). Foxp3^Cre^ x ST2^fl/fl^ mice (B6.Il1rl1^tm1c(KOMP)Wtsi^xFoxp3^tm1(cre)Saka^) (21) were provided by Astrid M. Westendorf (Infection Immunology, Institute of Medical Microbiology, University Hospital Essen, University of Duisburg-Essen, Essen, Germany). C57BL/6 mice, FIR-tiger (Foxp3-IRES-mRFP [FIR]xIL-10-IRES-GFP [tiger]) (22) and all other transgenic mice were bred in the animal facility of the University Medical Center Hamburg-Eppendorf (Hamburg, Germany) according to the Federation of European Laboratory Animal Science Association guidelines. All mice received human care according to the criteria outlined in the “Guide for the Care and Use of Laboratory Animals”. The experiments were approved by the institutional review board (Behörde für Justiz und Verbraucherschutz, Hamburg, Germany) and carried out according to the German animal protection law. Mice were housed in IVC cages under controlled conditions (22°C, 55% humidity, and 12-hour day-night rhythm) and fed a standard laboratory chow (LASvendi, Altromin, Germany). We have used male mice for the *in vivo* and a mixture of male and female mice for the *in vitro* studies.

### Animal treatment

Mice intraperitoneally received recombinant murine (rm)IL-33 (0.3 µg/mouse; BioLegend, San Diego, CA) on three or four consecutive days. To induce immune-mediated hepatitis, ConA (7 mg/kg; Sigma-Aldrich, München, Germany) was intravenously injected and mice were analyzed 24 hours later. For adoptive transfer experiments, FIR-tiger mice were treated with rmIL-33 (0.3 µg/mouse) or PBS on three consecutive days. Thereafter, Foxp3^+^ (mRFP^+^) CD4^+^ Tregs were isolated by FACS. 3x10^5^ Tregs were transferred into C57BL/6 recipient mice, which received ConA (7 mg/kg) 24 hours later. Mice were analyzed one day after induction of hepatitis. Heart blood was drawn from individual mice and liver injury was quantified by automated measurement of plasma activities of alanine transaminase (ALT) using a COBAS Mira System (Roche Diagnostic, Mannheim, Germany).

### Immunohistochemistry

Liver samples were fixed with 4% formalin and embedded in paraffin. 3 μm liver sections were cut, stained with hematoxylin and eosin (H&E) following standard protocols, and analyzed by light microscopy.

### Isolation of hepatic ILC2s

To expand hepatic ILC2s for isolation, C57BL/6 mice were treated with rmIL-33 (0.3 µg/mouse) on four consecutive days. Hepatic non-parenchymal cells were isolated by Percoll density gradient centrifugation. Single non-parenchymal cells were stained with lineage antibodies (anti-CD3, 145-2C11; anti-CD11b, M1/70; anti-CD45R/B220, RA3-6B2; anti-TER-119, TER-119; anti-GR1, RB6-8C5; all APC; all BioLegend), anti-Sca-1 (D7, Pacific Blue) and anti-ST2 (RMST2-2, PE-Cy7; both BioLegend) antibodies. Lineage-negative (lin^-^) cells were enriched by MACS and ST2^+^ Sca-1^+^ lin^-^ ILC2s were isolated by FACS (BD FACSAriaTM III sorter; BD Biosciences, Heidelberg, Germany) (23).

### Isolation of CD4^+^ T cells and CD25^+^ CD4^+^ Tregs

CD4^+^ T cells were isolated from spleen and lymph nodes using the CD4^+^ T Cell Isolation Kit (Miltenyi Biotec, Bergisch Gladbach, Germany) according to the manufacturer’s instructions. For Treg isolation, CD4^+^ T cells were stained with an anti-CD25-PE antibody (PC61, BioLegend) and anti-PE MicroBeads (Miltenyi Biotec), and CD25^+^ CD4^+^ Tregs were isolated by MACS.

### Immune cell culture

2x10^4^ CD25^+^ CD4^+^ Tregs were cultured with rmIL-2 (100 U/ml; R&D Systems, Minneapolis, MN) in presence of rmIL-33 (10 ng/ml; BioLegend), rmAREG (10 ng/ml; R&D Systems) or both (10 ng/ml each) for 1.5 days. 1.5x10^4^ hepatic ILC2s were cultured with rmIL-2 (1 U/ml) in presence of rmIL-33 (10 ng/ml), rmAREG (10 ng/ml) or both (10 ng/ml each) for 3.5 days.

### Suppression assay

CD4^+^ T cells were isolated from spleen and lymph nodes of C57BL/6 mice and stained with an anti-CD25-PE antibody and anti-PE MicroBeads. CD25^-^ CD4^+^ responder T cells were isolated by MACS and labeled with carboxyfluorescein succinimidyl ester (CFSE; CFSE Cell Division Tracker Kit, Biolegend) to assess T-cell proliferation. CD25^+^ CD4^+^ Tregs were isolated from spleen and lymph nodes of C57BL/6 and *Areg* ^-/-^ mice by MACS. 1x10^5^ responder CD4^+^ T cells were cultured with 1x10^5^ Tregs for 2.5 days. For responder CD4^+^ T-cell activation, co-cultures were done with Dynabeads (Dynabeads Mouse T-Activator CD3/CD28; ThermoFisher Scientific).

The percentage of inhibition was calculated as $\frac{\left(highest\;value\;\%proliferated\;cells-x\right)}{highest\;value\;\%proliferated\;cells}\times 100$ .

### Flow cytometry

Cells were incubated with anti-CD16/32 antibody (93; BioLegend) prior to antibody staining in order to prevent unspecific binding. LIVE/DEAD Fixable Staining Kits (Thermo Fisher Scientific) were used to exclude dead cells. For cell surface analysis, cells were stained with fluorochrome-labelled antibodies listed in **Supplementary Table S1**. For intracellular and intranuclear staining, cells were re-stimulated with phorbol myristate acetate (20 ng/ml) and ionomycin (1 µg/ml) for 4 hours with the addition of brefeldin A (1 µg/ml; all Sigma Aldrich) and monensin (2 µM; BioLegend) after 30 min. Thereafter, cells were fixed using the Transcription Factor Staining Buffer Set (ThermoFisher Scientific, Waltham, MA) and incubated in Permeabilization buffer with antibodies listed in **Supplementary Table S2**. Data were acquired using a BD LSRFortessa II (BD Biosciences) and analyzed by FlowJo software (Tree Star, Ashland, OR, USA).

### ELISA

Plasma AREG levels were analyzed using the Mouse Amphiregulin ELISA DuoSets (R&D Systems) and the Infinite M200 plate reader (Tecan, Männedorf, Switzerland) according to the manufacturer’s instructions.

### Quantitative real-time PCR analysis

Total RNA was isolated from shock-frozen liver tissue using the NucleoSpin RNA Midi Kit (Macherey-Nagel, Düren, Germany). Genomic DNA was digested using the DNA-*free* Kit (ThermoFisher Scientific). 1 µg RNA was transcribed into cDNA using the Verso cDNA Synthesis Kit (ThermoFisher Scientific) and the Biometra TAdvanced thermal cycler (Analytik Jena, Jena, Germany). Quantitative RT-PCR was performed using the PowerUp SYBR Green Master Mix (ThermoFisher Scientific) and the QuantStudio 7 Flex (Applied Biosystems, Waltham, USA). Relative mRNA levels were calculated using the ΔΔCT method after normalization to the reference gene β-actin. Quantification was shown in x-fold changes to the corresponding control cDNA. Primers were designed for detection of exon overlapping amplicons and were obtained from Metabion (Martinsried, Germany). Sequences of the primers are listed in **Supplementary Table S3**.

### Statistical analysis

Data were analyzed using the GraphPad Prism software (GraphPad software, San Diego, CA). For comparisons between two groups, a non-parametric Mann-Whitney U test and for more than two groups, a one-way ANOVA with Tukey’s *post-hoc* test were used. Data were shown as medians. A p value of less than 0.05 was considered statistically significant with the following ranges *p< 0.05, **p< 0.01, ***p< 0.001, and ****p< 0.0001.

## Results

### Strong activation of ST2^+^ Tregs in immune-mediated hepatitis

**Figure 1:**
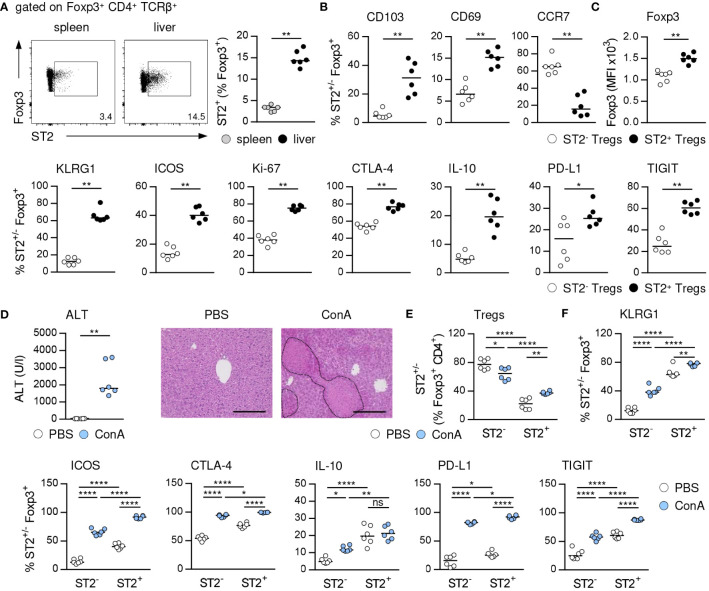
Phenotype of ST2^+^ Foxp3^+^ Tregs in liver homeostasis and immune-mediated hepatitis. **(A)** Frequency of ST2^+^ Tregs was determined in liver and spleen of naïve WT mice. Representative dot plots are shown. **(B, C)** Phenotype of hepatic ST2^+^ and ST2^-^ Tregs was analyzed in naïve WT mice. **(D)** WT mice received ConA and were analyzed 24 hours later. Plasma ALT levels were determined. Liver sections were stained with H&E to visualize necrotic areas (dotted line). **(E)** Frequency and **(F)** phenotype of hepatic ST2^+^ and ST2^-^ Tregs were analyzed. Bars represent 200 µm. Medians of two independent experiments with 3 mice per group and experiment are shown. *p< 0.05; **p< 0.01; ****p< 0.0001; ns, not significant; MFI, mean fluorescent intensity.

We analyzed the phenotype of hepatic ST2^+^ Foxp3^+^ Tregs in immune-mediated hepatitis to assess their impact on liver disease pathology. ST2^+^ Tregs have been described as a tissue-associated Treg subset crucial for immunosuppression and organ homeostasis (10, 24). In line with this, we determined an elevated frequency of ST2^+^ Foxp3^+^ Tregs in naïve liver compared to spleen (**Figure 1A**, **Supplementary Figure 1A**). Hepatic ST2^+^ Foxp3^+^ Tregs showed enhanced expression of the tissue residency markers CD103 and CD69 compared to ST2^-^ Foxp3^+^ Tregs, while they expressed less CCR7, a chemokine receptor required for lymph node migration (**Figure 1B**, **Supplementary Figure 1B**). Phenotypically, hepatic ST2^+^ Foxp3^+^ Tregs showed stronger expression of their key transcription factor Foxp3, the activation marker killer cell lectin like receptor G1 (KLRG1) and inducible T cell co-stimulator (ICOS), of the proliferation marker Ki-67, and of the inhibitory molecules cytotoxic T-lymphocyte-associated protein 4 (CTLA-4), IL-10, programmed death ligand 1 (PD-L1) and T cell immunoreceptor with Ig and ITIM domains (TIGIT) than ST2^-^ Foxp3^+^ Tregs in liver homeostasis (**Figures 1C**, **Supplementary Figure 1B**). In immune-mediated hepatitis, liver injury and formation of necrotic lesions (**Figure 1D**) were associated with an elevated frequency of hepatic ST2^+^ Foxp3^+^ Tregs (**Figure 1E**). Expression of Foxp3 was not further increased in ST2^+^ and ST2^-^ Foxp3^+^ Tregs in liver inflammation (**Supplementary Figure 1C**). Both, hepatic ST2^+^ and ST2^-^ Tregs were activated in immune-mediated hepatitis and enhanced expression of inhibitory molecules. In fact, by calculating the fold change in frequencies, we found stronger activation of ST2^-^ Tregs compared to ST2^+^ Tregs in immune-mediated hepatitis (KLRG1: ST2^-^ 3.4 ± 1.0, ST2^+^ 1.2 ± 0.1; ICOS: ST2^-^ 4.9 ± 1.2, ST2^+^ 2.3 ± 0.2). Expression of inhibitory molecules was also stronger induced in ST2^-^ Tregs (CTLA-4: ST2^-^ 1.7 ± 0.2, ST2^+^ 1.3 ± 0.1; IL-10: ST2^-^ 2.5 ± 0.9, ST2^+^ 1.1 ± 0.5; PD-L1: ST2^-^ 9.7 ± 8.8, ST2^+^ 3.6 ± 0.7; TIGIT: ST2^-^ 2.3 ± 0.8, ST2^+^ 1.5 ± 0.1). We believe that due to the elevated basal activation and inhibitory molecule expression of ST2^+^ Tregs, they do not become activated as strong as ST2^-^ Tregs in immune-mediated hepatitis. However, activation and inhibitory molecule expression were still elevated in ST2^+^ compared to ST2^-^ Tregs (**Figure 1F**). Thus, ST2^+^ Foxp3^+^ Tregs constituted an activated effector Treg subset in the murine liver.

### Increased liver injury in absence of ST2^+^ Tregs

**Figure 2:**
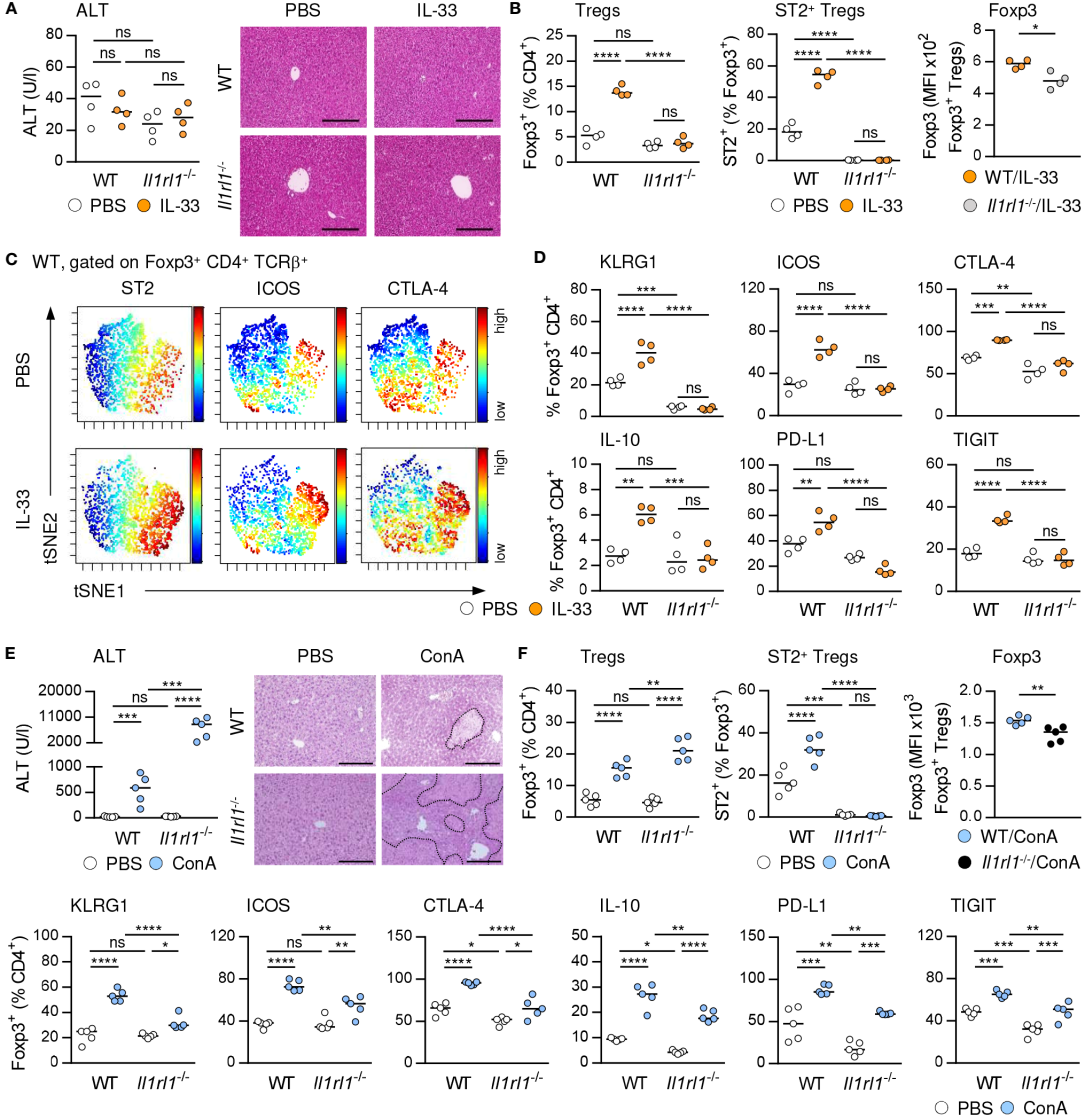
IL-33-induced activation of hepatic ST2^+^ Foxp3^+^ Tregs. **(A)** WT and *Il1rl1* ^-/-^ mice were treated with IL-33 on four consecutive days. Plasma ALT levels were determined. Liver sections were stained with H&E. **(B)** Frequencies and MFI of Foxp3 in hepatic Tregs were analyzed. **(C)** ViSNE analysis of Tregs from WT mice is shown. **(D)** Phenotype of Tregs from WT and *Il1rl1* ^-/-^ mice was analyzed. **(E)** WT and *Il1rl1* ^-/-^ mice received ConA and were analyzed 24 hours later. Plasma ALT levels were determined. Liver sections were stained with H&E to visualize necrotic areas (dotted line). **(F)** Frequency and phenotype of hepatic Tregs were analyzed. Bars represent 200 µm. Medians of one out of two independent experiments with 4-5 mice per group and experiment are shown. *p< 0.05; **p< 0.01; ***p< 0.001; ****p< 0.01; ns, not significant; MFI, mean fluorescent intensity.

IL-33 is the only known ligand of ST2 and has been described as a potent activator of Tregs (4). We demonstrated that treatment with exogenous IL-33, which did not induce liver injury and tissue damage (**Figure 2A**), selectively increased the frequency of hepatic ST2^+^ Foxp3^+^ Tregs in C57BL/6 wild-type (WT) mice and their activation and expression of inhibitory molecules. ST2^+^ Foxp3^+^ Tregs did not enhance expression of Foxp3 in response to exogenous IL-33. We determined elevated Foxp3 expression by Foxp3^+^ Tregs from IL-33-treated WT compared to *Il1rl1* ^-/-^ mice, probably due to lack of ST2^+^ Tregs in *Il1rl1* ^-/-^ mice (**Figures 2B, C**, **Supplementary Figure 2A**). Moreover, Foxp3^+^ Tregs of IL-33-treated WT mice were stronger activated and showed enhanced expression of inhibitory molecules than Foxp3^+^ Tregs from IL-33-treated *Il1rl1* ^-/-^ mice (**Figure 2D**). Thus, targeting the IL-33/ST2 axis by application of exogenous IL-33 selectively induced ST2^+^ Treg activation and expansion in the liver. In addition, culture of WT Tregs in presence of IL-33 enhanced their expansion, activation and expression of inhibitory molecules, whereas Tregs from *Il1rl1* ^-/-^ mice were not stimulated by IL-33 *in vitro*. Similar to the *in vivo* data, IL-33-stimulated *Il1rl1* ^-/-^ Foxp3^+^ Tregs showed decreased Foxp3 expression compared to WT Foxp3^+^ Tregs *in vitro* (**Supplementary Figures 2B, C**). We further showed that ST2^+^ but not ST2^-^ Foxp3^+^ Tregs from WT mice were activated by IL-33 *in vitro* (**Supplementary Figure 2D**).

We and others have shown that ST2-deficient (*Il1rl1* ^-/-^) mice developed more severe liver injury and tissue damage than WT mice (**Figure 2E**) (20), suggesting an immunoregulatory role of endogenous IL-33 in immune-mediated hepatitis. Liver pathology was aggravated in *Il1rl1* ^-/-^ mice despite the absence of hepatic ILC2s (**Supplementary Figure 3**) and an elevated frequency of Foxp3^+^ Tregs in the inflamed liver (**Figure 2F**). However, *Il1rl1* ^-/-^ mice lacked ST2^+^ Foxp3^+^ Tregs and the remaining Foxp3^+^ Treg population showed reduced Foxp3 expression, activation and expression of inhibitory molecules in immune-mediated hepatitis compared to Foxp3^+^ Tregs in WT mice (**Figure 2F**). Thus, endogenous IL-33 selectively activated hepatic ST2^+^ Foxp3^+^ Tregs and lack of this Treg subset in *Il1rl1* ^-/-^ mice resulted in an overall reduced Treg activation in immune-mediated hepatitis.

### IL-33-activated Tregs suppressed immune-mediated hepatitis

**Figure 3:**
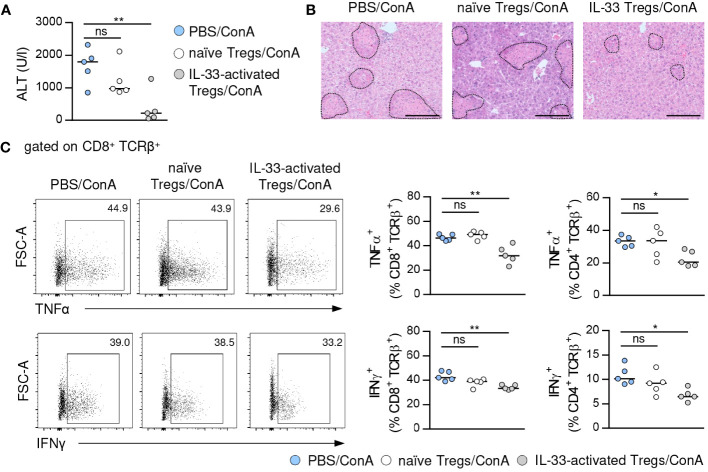
Immunosuppressive potential of IL-33-activated Tregs. **(A)** FIR-tiger mice were treated with IL-33 or PBS on three consecutive days. Foxp3^+^ Tregs were isolated by FACS and transferred into WT mice one day before ConA challenge. WT mice were analyzed 24 hours after induction of immune-mediated hepatitis. Plasma ALT activity was determined. **(B)** Liver samples were stained with H&E to visualize necrotic areas (dotted line). **(C)** Phenotype of hepatic CD4^+^ and CD8^+^ T cells was analyzed. Representative dot plots are shown. Bars represent 200 µm. Medians of one out of two independent experiments with 5 mice per group and experiment are shown. *p< 0.05; **p< 0.01; ns, not significant.

To assess the immunosuppressive capacity of Tregs activated by exogenous IL-33, we isolated Foxp3^+^ Tregs from IL-33-treated FIR-tiger mice and adoptively transferred them into WT mice one day before induction of immune-mediated hepatitis. As control, naïve Foxp3^+^ Tregs from PBS-treated FIR-tiger mice were transferred. We showed reduced liver injury and formation of necrotic lesions after transfer of IL-33-activated Foxp3^+^ Tregs (**Figures 3A, B**). This was associated with decreased expression of the pro-inflammatory cytokines interferon (IFN)γ and tumor necrosis factor (TNF)α by hepatic CD8^+^ and CD4^+^ T cells (**Figure 3C**). In contrast, transfer of naïve Foxp3^+^ Tregs did not alter disease pathology and immune cell phenotype (**Figures 3A-C**), demonstrating the high immunosuppressive potential of Tregs that were pre-activated through exogenous IL-33 in immune-mediated hepatitis.

### More severe immune-mediated hepatitis in absence of AREG

IL-33 has been described to induce AREG expression in Tregs and ILC2s thereby supporting Treg function and tissue repair (6, 12). Indeed, we showed increased AREG expression in hepatic ST2^+^ Foxp3^+^ Tregs and ILC2s upon treatment with exogenous IL-33 (**Supplementary Figure 4A**). IL-33 also enhanced expression of AREG in ST2^+^ Foxp3^+^ Tregs (**Supplementary Figure 4B**) and hepatic ILC2s (**Supplementary Figures 5A, B**) *in vitro*.

**Figure 4:**
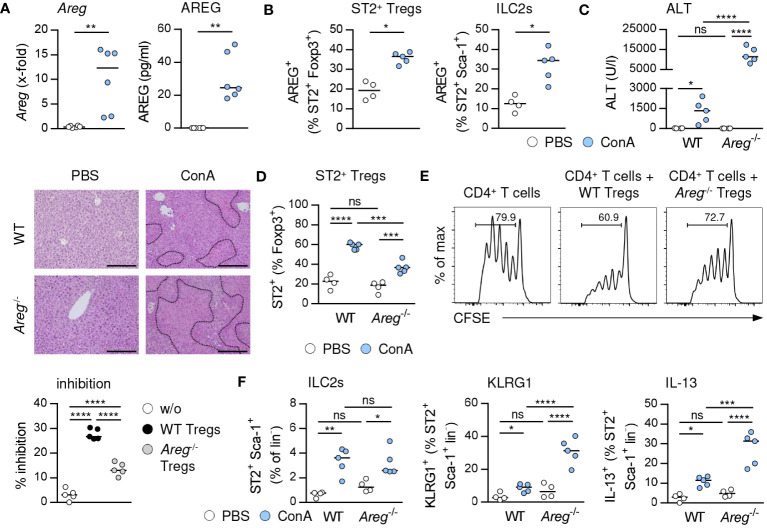
Immunoregulatory role of AREG in immune-mediated hepatitis. **(A)** WT and *Areg* ^-/-^ mice received ConA and were analyzed 24 hours later. *Areg* mRNA expression and plasma AREG levels were analyzed in WT mice. **(B)** AREG expression was determined in hepatic ST2^+^ Tregs and ILC2s. **(C)** Plasma ALT levels were determined. Liver samples were stained with H&E to visualize necrotic areas (dotted line). **(D)** Frequency of hepatic ST2^+^ Tregs was analyzed. **(E)** CFSE-labeled CD25^-^ CD4^+^ T cells were activated in presence or absence of CD25^+^ CD4^+^ Tregs from WT and *Areg* ^-/-^ mice. Histograms show frequencies of proliferating CD4^+^ T cells. Percent inhibition of CD4^+^ T cell proliferation is depicted. **(F)** Frequency and phenotype of hepatic ILC2s were analyzed in WT and *Areg* ^-/-^ mice. Bars represent 200 µm. Medians of one out of two independent experiments with 4-5 mice per group and experiment are shown. *p< 0.05; **p< 0.01; ***p< 0.001; ****p< 0.01; ns, not significant; CSFE, carboxyfluorescein succinimidyl ester; MFI, mean fluorescent intensity.

In immune-mediated hepatitis, liver *Areg* mRNA and serum levels of AREG were elevated (**Figure 4A**), and we determined enhanced expression of AREG in hepatic ST2^+^ Foxp3^+^ Tregs and ILC2s (**Figure 4B**). In *Il1rl1* ^-/-^ mice, Foxp3^+^ Tregs showed reduced expression of AREG compared to Foxp3^+^ Tregs from WT mice, suggesting that endogenous IL-33 induced AREG expression in ST2^+^ Tregs in liver inflammation (**Supplementary Figure 4C**). In *Areg* ^-/-^ mice, ST2^+^ Foxp3^+^ Tregs and ILC2s did not express AREG (**Supplementary Figure 4C**). Interestingly, *Areg* ^-/-^ mice developed more severe liver injury and tissue damage than WT mice (**Figure 4C**). Hepatic *Il33* mRNA expression was not altered in *Areg* ^-/-^ compared to WT mice in immune-mediated hepatitis (**Supplementary Figure 4C**), indicating that the elevated liver injury shown in *Areg* ^-/-^ mice did not correlate with increased *Il33* mRNA induction. Lack of AREG resulted in a reduced frequency of hepatic ST2^+^ Foxp3^+^ Tregs in immune-mediated hepatitis (**Figure 4D**), whereas their phenotype was not altered compared to ST2^+^ Foxp3^+^ Tregs from WT mice. Notably, *Areg* ^-/-^ ST2^+^ Foxp3^+^ Tregs up-regulated expression of Foxp3 and the anti-apoptotic protein B cell lymphoma 2 (Bcl-2) during liver inflammation (**Supplementary Figure 4D**), probably to increase their stability in absence of AREG. Functionally, we showed an impaired capacity of *Areg* ^-/-^ CD25^+^ Tregs to inhibit activation and subsequent proliferation of CD25^-^ CD4^+^ T cells *in vitro* (**Figure 4E**).

The frequency of hepatic ST2^+^ Foxp3^+^ Tregs was also reduced in *Areg* ^-/-^ mice treated with exogenous IL-33. Again, their phenotype was comparable to ST2^+^ Foxp3^+^ Tregs from IL-33-treated WT mice, but they showed elevated expression of Foxp3 as well as Ki-67 (**Supplementary Figure 4E**), indicating stronger proliferation. *In vitro*, IL-33 induced a lower expansion of ST2^+^ Foxp3^+^ Tregs from *Areg* ^-/-^ mice, and the IL-33-induced expression of inhibitory molecules was reduced in *Areg* ^-/-^ compared to WT ST2^+^ Foxp3^+^ Tregs (**Supplementary Figure 4F**).

We further demonstrated similar frequencies of hepatic ILC2s in *Areg* ^-/-^ and WT mice in immune-mediated hepatitis. However, lack of AREG resulted in an enhanced ILC2 activation and IL-13 expression (**Figure 4F**). In contrast, activation and type 2 cytokine expression of ILC2s were not altered in *Areg* ^-/-^ compared to WT mice upon treatment with exogenous IL-33 (**Supplementary Figure 5C**). Thus, AREG exerted an immunoregulatory function in immune-mediated hepatitis.

### Antagonistic effects of exogenous AREG on ST2^+^ Tregs and ILC2s

**Figure 5:**
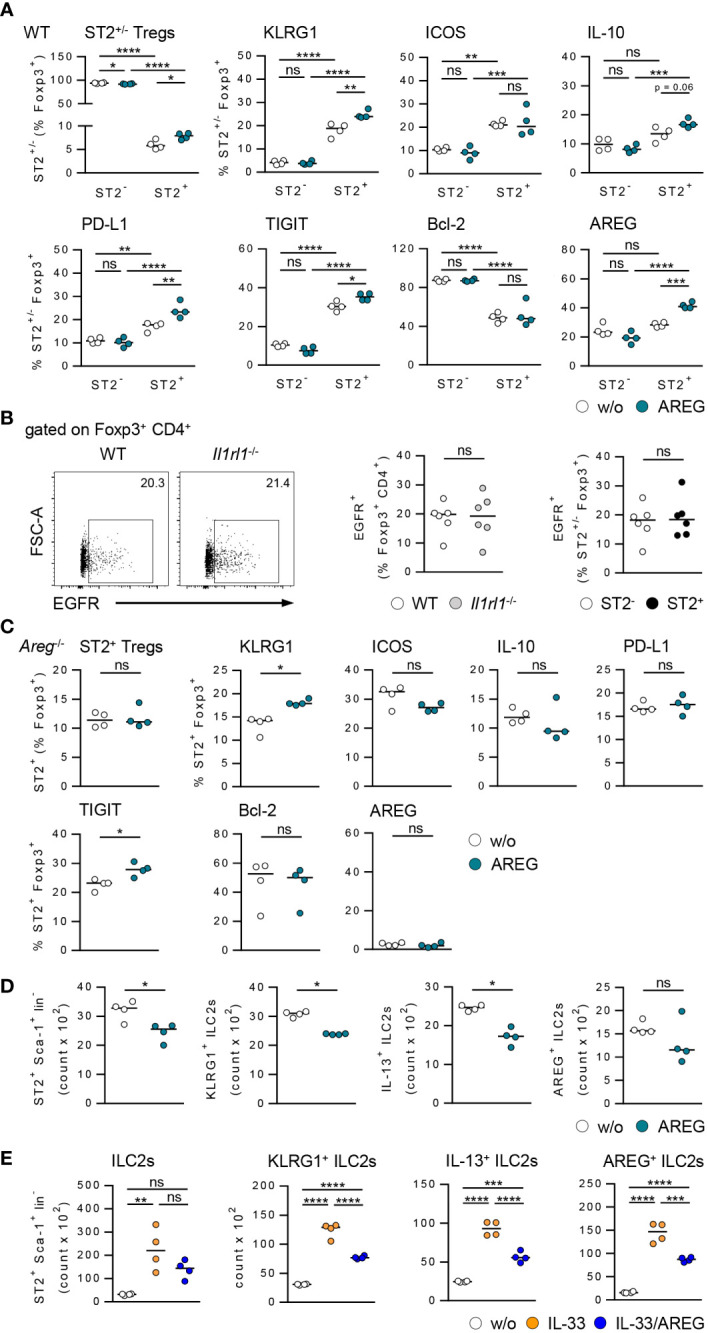
Effects of AREG on activation of ST2^+^ Foxp3^+^ Tregs and ILC2s *in vitro*. **(A)** CD25^+^ CD4^+^ Tregs were isolated from WT mice and cultured in presence of AREG for 1.5 days. Phenotype of ST2^+^ and ST2^-^ Tregs was analyzed. **(B)** EGFR expression was determined in Foxp3^+^ Tregs from WT and *Il1rl1* ^-/-^ mice as well as ST2^+^ and ST2^-^ Tregs from WT mice. 6 mice per group from two individual experiments are shown. **(C)** CD25^+^ CD4^+^ Tregs were isolated from *Areg* ^-/-^ mice and cultured in presence of AREG for 1.5 days. Phenotype of ST2^+^ Tregs was analyzed. **(D, E)** Hepatic ILC2s were isolated from IL-33-treated WT mice and cultured in presence of **(D)** AREG and **(E)** IL-33 or IL-33/AREG for 3.5 days. ILC2 number and phenotype were determined. Medians of one out of two independent experiments are shown. 4 wells per sample and experiment were seeded into culture plates. *p< 0.05; **p< 0.01; ***p< 0.001; ****p< 0.01; ns, not significant; w/o, without.

To further assess the effect of AREG on immune cell phenotype, we cultured Tregs and hepatic ILC2s in presence of AREG. Exogenous AREG induced an activation and expansion of ST2^+^ Foxp3^+^ Tregs. They increased expression of inhibitory molecules, whereas Bcl-2 expression was not altered. Moreover, AREG stimulated its own expression in ST2^+^ Foxp3^+^ Tregs. In contrast, ST2^-^ Foxp3^+^ Tregs were not activated by AREG (**Figure 5A**). We did also not observe an effect of AREG on activation of Foxp3^+^ Tregs from *Il1rl1* ^-/-^ mice (**Supplementary Figure 6A**), demonstrating that exogenous AREG selectively activated ST2^+^ Foxp3^+^ Tregs.

Since AREG is a ligand of the epidermal growth factor receptor (EGFR), we analyzed EGFR expression by Tregs from WT and *Il1rl1* ^-/-^ mice. The frequency of EGFR^+^ Foxp3^+^ Tregs was not altered in *Il1rl1* ^-/-^ compared to WT mice. ST2^+^ and ST2^-^ Foxp3^+^ Tregs from WT mice did also not differ in their EGFR expression (**Figure 5B**), indicating an EGFR-independent effect of AREG on ST2^+^ Foxp3^+^ Tregs *in vitro*. We also showed that exogenous AREG did not induce an expansion of ST2^+^ Foxp3^+^ Tregs from *Areg* ^-/-^ mice. With the exception of TIGIT, AREG-deficient ST2^+^ Foxp3^+^ Tregs did not up-regulate expression of inhibitory molecules or Bcl-2 in response to AREG (**Figure 5C**).

Culture of hepatic ILC2s in presence of AREG resulted in a reduced ILC2 number and decreased their activation and IL-13 expression (**Figure 5D**). Moreover, exogenous AREG reduced the IL-33-induced activation of ILC2s and their expression of IL-13 and AREG (**Figure 5E**). In contrast, AREG did not affect the activation of WT or *Areg* ^-/-^ ST2^+^ Foxp3^+^ Tregs by IL-33 *in vitro* (**Supplementary Figures 6B, C**). Thus, exogenous AREG differentially regulated the activation of ST2^+^ Foxp3^+^ Tregs and hepatic ILC2s *in vitro*.

### ST2^+^ Treg activation is critical for IL-33-induced immunosuppression

**Figure 6:**
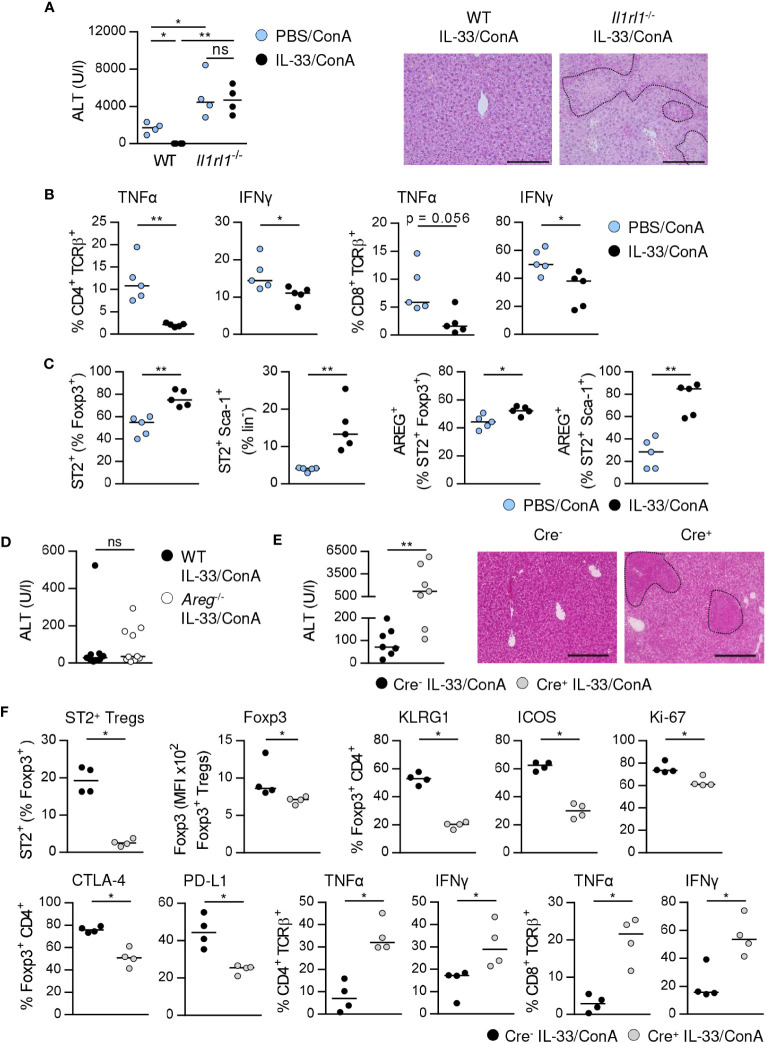
ST2^+^ Treg-dependent immunosuppressive effect of IL-33 on liver disease pathology. **(A)** WT and *Il1rl1* ^-/-^ mice were treated with IL-33 on three consecutive days. On the fourth day, mice received ConA and were analyzed 24 hours later. Plasma ALT levels were determined. Liver sections were stained with H&E to visualize necrotic areas (dotted line). **(B)** Expression of TNFα and IFNγ by hepatic CD4^+^ and CD8^+^ T cells was determined. **(C)** Frequencies of hepatic ST2^+^ Foxp3^+^ Tregs and ILC2s and their expression of AREG were analyzed. **(D)** WT and *Areg* ^-/-^ mice were treated with IL-33 for three days. At day four, mice received ConA and were analyzed one day later. Plasma ALT levels were determined. 9-10 mice per group from two individual experiments are shown. **(E)** Foxp3^Cre+^ x ST2^fl/fl^ and Foxp3^Cre-^ x ST2^fl/fl^ mice were treated with IL-33 for three days. At day four, mice received ConA and were analyzed 24 hours later. ALT levels were determined. Liver sections were stained with H&E to visualize necrotic areas (dotted line). **(F)** Frequency of hepatic ST2^+^ Foxp3^+^ Tregs was determined. Phenotype of hepatic Foxp3^+^ Tregs and CD4^+^ and CD8^+^ T cells was analyzed. Bars represent 200 µm. Medians of one out of two independent experiments with 4-5 mice per group and experiment are shown. *p< 0.05; **p< 0.01; ns, not significant; MFI, mean fluorescent intensity.

Previously, we have shown that treatment of WT mice with exogenous IL-33 before ConA challenge potently suppressed immune-mediated hepatitis (17). In contrast, liver injury and tissue damage were not improved in *Il1rl1* ^-/-^ mice (**Figure 6A**), demonstrating that targeting the IL-33/ST2 axis by exogenous IL-33 ameliorated disease pathology. In WT mice, we observed reduced frequencies of hepatic CD4^+^ and CD8^+^ T cells expressing IFNγ and TNFα in immune-mediated hepatitis after pre-treatment with exogenous IL-33 (**Figure 6B**). The frequencies of hepatic ST2^+^ Foxp3^+^ Tregs and ILC2s were increased and both up-regulated expression of AREG (**Figure 6C**). Moreover, Foxp3 expression and activation of ST2^+^ Foxp3^+^ Tregs but not their expression of inhibitory molecules was further enhanced by treatment with exogenous IL-33 (**Supplementary Figure 7**).

We analyzed whether exogenous IL-33 mediates its immunosuppressive effect via induction of AREG. Therefore, we treated *Areg* ^-/-^ mice with IL-33 before ConA challenge. However, ALT levels were not significantly altered in *Areg* ^-/-^ compared to WT mice (**Figure 6D**). To assess whether activation of ST2^+^ Tregs by exogenous IL-33 is critical for the immunosuppressive effect of this cytokine on liver disease pathology, we treated Foxp3^Cre^ x ST2^fl/fl^ mice (21) with IL-33 before induction of hepatitis. Foxp3^Cre+^ x ST2^fl/fl^ mice developed more severe liver injury and tissue damage than Foxp3^Cre-^ x ST2^fl/fl^ mice after treatment with exogenous IL-33 (**Figure 6E**). This correlated with a strongly reduced frequency of hepatic ST2^+^ Tregs in Foxp3^Cre+^ x ST2^fl/fl^ mice compared to Foxp3^Cre-^ x ST2^fl/fl^ mice (**Figure 6F**). Moreover, we showed decreased Foxp3 expression, activation, proliferation and inhibitory molecule expression by hepatic Foxp3^+^ Tregs as well as elevated expression of TNFα and IFNγ by hepatic CD4^+^ and CD8^+^ T cells in Foxp3^Cre+^ x ST2^fl/fl^ mice in immune-mediated hepatitis after pre-treatment with IL-33 (**Figure 6F**). Thus, the immunosuppressive effect of exogenous IL-33 depends on ST2^+^ Treg activation.

## Discussion

**Figure 7:**
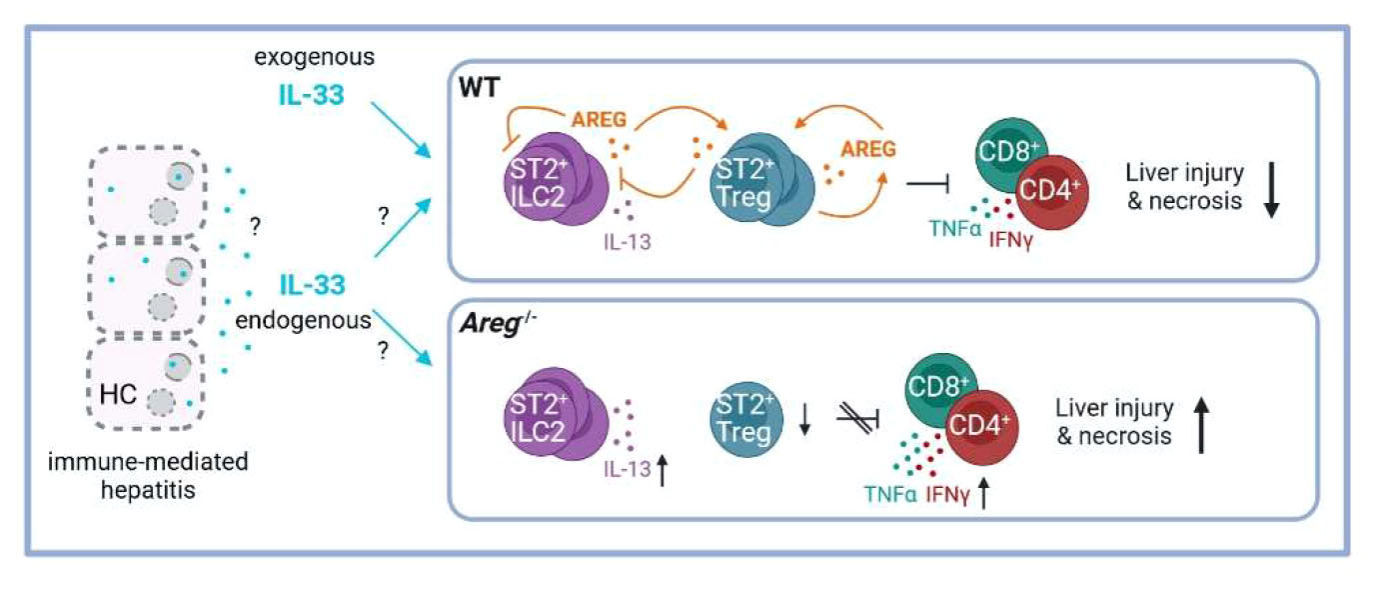
Scheme of the proposed ST2^+^ Treg/AREG axis in immune-mediated hepatitis. IL-33 induces activation of hepatic ST2^+^ Tregs and ILC2s, as well as their expression of AREG in immune-mediated hepatitis. Paracrine AREG further increases activation and AREG production of ST2^+^ Tregs, while suppressing activation and effector cytokine expression by hepatic ILC2s. Disease pathology of immune-mediated hepatitis was aggravated in absence of AREG, which correlated with enhanced ILC2 activation and effector cytokine expression and reduced ST2^+^ Tregs in the inflamed liver. Created with BioRender.com. HC, hepatocytes.

Immune-mediated liver diseases such as AIH are live-threatening diseases with limited therapeutic options so far. Thus, understanding cellular and molecular mechanisms in hepatic inflammation will provide the opportunity to selectively target pathways involved in disease pathology. In this study, we described the immunoregulatory role of AREG in immune-mediated hepatitis by differentially affecting two liver-residing immune cell populations, ST2^+^ Tregs and ILC2s. The main findings are summarized in **Figure 7**.

Our Treg phenotype analysis revealed ST2^+^ Tregs as an activated Treg subset residing in the murine liver, which was characterized by higher expression of the Treg-defining transcription factor Foxp3 and inhibitory molecules crucial for immunosuppressive function than ST2^-^ Tregs. This is in line with other studies describing ST2^+^ Tregs as a tissue-associated effector Treg subset (9, 10). Moreover, ST2^+^ Tregs were shown to be superior to ST2^-^ Tregs in suppressing CD4^+^ T-cell activation *in vitro* through enhanced expression of inhibitory molecules (9). In immune-mediated hepatitis, ST2^+^ and ST2^-^ Tregs were activated, however, activation and inhibitory molecule expression was further elevated in ST2^+^ Tregs in liver inflammation. Evidence for an immunosuppressive function of ST2^+^ Tregs and their activation through endogenous IL-33 in hepatic inflammation derived from *Il1rl1* ^-/-^ mice, which developed more severe immune-mediated hepatitis despite an elevated frequency of Tregs in the liver. However, due to lack of ST2^+^ Tregs in *Il1rl1* ^-/-^ mice, the remaining Tregs were less activated and showed decreased expression of inhibitory molecules in immune-mediated hepatitis. Another study also describing increased liver injury in *Il1rl1* ^-/-^ mice showed enhanced T-cell numbers and IFNγ expression (20), which might be a consequence of impaired Treg-mediated suppression of inflammatory T-cell responses in mice lacking ST2^+^ Tregs. In addition, one study using *Il33* ^-/-^ mice correlated impaired activation of ST2^+^ Tregs in absence of endogenous IL-33 with enhanced hepatic infiltration of NK cells and an aggravated liver injury (19). Taken together, these studies highlight the immunoregulatory role of endogenous IL-33 in immune-mediated hepatitis through inhibiting inflammatory immune cell activation and recruitment. Nevertheless, divergent effects of the IL-33/ST2 axis have been described in immune-mediated hepatitis. We have previously demonstrated that hepatic release of IL-33 induced activation of ILC2s that aggravated liver injury by recruitment of inflammatory eosinophils (17). Invariant natural killer T (iNKT) cells also became activated in immune-mediated hepatitis and induced strong nuclear IL-33 expression in hepatocytes (16, 25). Blockage of endogenous IL-33 signaling through an anti-IL-33 antibody reduced iNKT-cell activation and ameliorated disease pathology further supporting a pro-inflammatory role of IL-33 in immune-mediated hepatitis (18). Endogenous IL-33 has also been identified as a disease-driving factor in liver ischemia/reperfusion (I/R) injury, another disease with hepatic lesions. Here, *Il33* ^-/-^ (26, 27) and *Il1rl1* ^-/-^ mice (26) were shown to develop less severe liver I/R injury, which correlated with reduced infiltration of neutrophils (27) and formation of neutrophil extracellular traps in liver sinusoids (26). Moreover, iNKT cells have been implicated in the initiation of I/R injury and by using *Il33* ^-/-^ mice, it has been shown that their hepatic recruitment depended on release of IL-33 (28). Moreover, blockage of endogenous IL-33/ST2 signaling in *Il1rl1* ^-/-^ mice reduced acetaminophen-induced liver injury, in which massive necrosis and release of IL-33 correlated with hepatic recruitment of neutrophils (29). Thus, endogenous IL-33 aggravated hepatic I/R and drug-induced injury by recruitment of inflammatory immune cells to the liver. In contrast to the findings in *Il1rl1* ^-/-^ mice, one study demonstrated that IL-33 deficiency in *Il33* ^-/-^ mice exacerbated acetaminophen-induced liver injury by disrupting M2 macrophage polarization and activating hepatocyte autophagy. This demonstrates a protective effect of endogenous IL-33 in drug-induced liver injury through modulating macrophage polarization and hepatocyte autophagy (30). In conclusion, endogenous IL-33 can exert opposite effects on liver disease pathology depending on the targeted immune cell populations.

Our adoptive transfer experiments provided evidence for a superior immunosuppressive function of ST2^+^ Tregs activated by exogenous IL-33 in immune-mediated hepatitis and demonstrated an inhibitory effect on effector T-cell activation resulting in reduced expression of TNFα and IFNγ, two mediators of liver damage in immune-mediated hepatitis (31, 32). Since transfer of naïve Tregs did not ameliorate disease severity, this reveals the potential of exogenous IL-33 to selectively promote the anti-inflammatory effect of IL-33-responsive ST2^+^ Tregs in immune-mediated hepatitis. This is further supported by the finding that treatment with exogenous IL-33 protected Foxp3^Cre –^x ST2^fl/fl^ but not Foxp3^Cre+^ x ST2^fl/fl^ mice lacking ST2^+^ Tregs from immune-mediated hepatitis. Importantly, expression of TNFα and IFNγ by hepatic CD4^+^ and CD8^+^ T cells was enhanced in absence of ST2^+^ Tregs, further demonstrating that ST2^+^ Tregs pre-activated with exogenous IL-33 suppress inflammatory T-cell responses in immune-mediated hepatitis. Since one recent study demonstrated that Treg depletion resulted in enhanced iNKT-cell activation and liver injury in immune-mediated hepatitis, which was not the case in iNKT cell-deficient mice (33), it might conceivable that activated ST2^+^ Tregs also suppress inflammatory iNKT-cell activation.

The role of AREG in liver disease is not well understood. Elevated hepatic AREG expression have been described in patients with liver cirrhosis (34) and cholestatic liver disease (35). In mice, AREG were found to protect from cholestatic injury (35), and to promote liver regeneration after partial hepatectomy (34). In contrast, AREG aggravated murine liver fibrosis (36) and impaired anti-viral immune responses in murine hepatitis B virus infection (37). We here demonstrated a beneficial role of AREG in immune-mediated hepatitis, a well-established preclinical model that shares some similarities with acute AIH (15).

We focused our analyses to the acute inflammatory phase of the disease, where processes of liver regeneration have not started yet (38). In this disease phase, ST2^+^ Tregs and ILC2s, two liver-residing, IL-33-responsive immune cell subsets increased expression of AREG. That hepatic ILC2s can express AREG has also been shown in patients with various liver diseases (32). However, ST2^+^ Tregs and ILC2s were differentially influenced by AREG. ST2^+^ Tregs depended on intrinsic expression of AREG for their survival and function as shown by reduced ST2^+^ Treg numbers in *Areg* ^-/-^ mice despite enhanced expression of Foxp3 and anti-apoptotic Bcl-2 or stronger proliferation, and an impaired capacity of *Areg* ^-/-^ Tregs to suppress CD4^+^ T-cell activation. Interestingly, we found that exogenous AREG selectively activated ST2^+^ Tregs although ST2^-^ Tregs also expressed the AREG receptor EGFR. AREG has been described to promote the immunosuppressive activity of EGFR-expressing Tregs *in vitro* (12, 37). However, EGFR-independent mechanisms of AREG function need to be elucidated. Importantly, the activating effect of AREG was lost in *Areg* ^-/-^ ST2^+^ Tregs, indicating that exogenous AREG cannot compensate for the loss of intrinsic AREG expression in ST2^+^ Tregs. The finding of an impaired activation of *Areg* ^-/-^ ST2^+^ Tregs by exogenous IL-33 further highlights that AREG expression by ST2^+^ Tregs acts in an autocrine loop to promote their activation and function. Thus, exogenous IL-33 and AREG stimulate AREG expression in ST2^+^ Tregs thereby supporting their maintenance and function in immune-mediated hepatitis.

In contrast to the stimulating effect on ST2^+^ Tregs, exogenous AREG inhibited hepatic ILC2 activation and cytokine expression and also reduced IL-33-mediated activation of ILC2s *in vitro*. In line with this, we observed increased ILC2 activation and cytokine expression in immune-mediated hepatitis in absence of AREG. Thus, AREG-expressing ILC2s might support ST2^+^ Treg function and inhibit their own effector function in immune-mediated hepatitis. Since ST2^+^ Tregs also express AREG, they might contribute to the regulation of ILC2 activation in liver injury. Consistently, a stimulating effect of ILC2s on the suppressive capacity of Tregs has been shown *in vitro* (39). Moreover, Tregs were found to inhibit ILC2 cytokine expression *in vitro* and *in vivo* (40). Our data further showed that in contrast to ST2^+^ Tregs, ILC2s did not depend on intrinsic AREG expression for function since activation of ILC2s by exogenous IL-33 was not altered in *Areg* ^-/-^ mice.

AREG-expressing ST2^+^ Tregs and ILC2s may also exert a tissue protective effect in immune-mediated hepatitis as it was shown for Tregs in muscle repair (41) and viral infection (42) as well as for ILC2s in biliary atresia (43), lung infection (6) and intestinal inflammation (8). In the liver, AREG has been identified as mitogen for hepatocytes thereby supporting hepatic regeneration (34). A recent studies further showed that AREG promoted epithelial homeostasis and repair in experimental biliary atresia (43).

There are some limitations of the study. The functional relevance of AREG specifically expressed by ST2^+^ Tregs or ILC2s in immune-mediated hepatitis is still not known. Adoptive transfer experiments with Tregs or hepatic ILC2s isolated from *Areg* ^-/-^ mice would be possible to assess disease pathology of immune-mediated hepatitis if Tregs or hepatic ILC2s do not express AREG. Moreover, the effect of exogenous AREG on ST2^+^ Tregs and hepatic ILC2s has been investigated *in vitro*. To strengthen these data, treatment of mice with exogenous AREG and subsequent ST2^+^ Treg and ILC2 phenotype analysis could be done. Additionally, we compared frequencies of the different immune cells in our analyses. Cell numbers would support these data and therefore, counting beads should be included in the analyses to calculate cell numbers in future studies. In the end, this is a murine study, which allows functional analyses to identify novel immunological pathways involved in the pathogenesis of immune-mediated hepatitis. However, the findings presented here need to be further addressed in patients with immune-mediated liver disease.

In summary, this study reveals the high immunosuppressive capacity of ST2^+^ Tregs in immune-mediated hepatitis, which depend on intrinsic AREG as well as exogenous IL-33 for their maintenance, activation and function in liver inflammation. It further identifies AREG as inhibitor of hepatic ILC2 effector function. The fact that the ST2^+^ Treg/AREG axis promotes immunoregulation and inhibits immunity in immune-mediated hepatitis provides important insights into the cellular and molecular mechanisms through which hepatic immune responses are regulated in immune-mediated hepatitis.

## Data availability statement

The original contributions presented in the study are included in the article/**Supplementary Material**. Further inquiries can be directed to the corresponding author.

## Ethics statement

The animal study was approved by Behörde für Justiz und Verbraucherschutz, Hamburg, Germany. The study was conducted in accordance with the local legislation and institutional requirements.

## Author contributions

SW: Formal analysis, Investigation, Methodology, Writing – review & editing. FJ: Formal analysis, Investigation, Methodology, Writing – review & editing. AO: Investigation, Writing – review & editing. FH: Investigation, Writing – review & editing. AW: Methodology, Writing – review & editing. GT: Conceptualization, Writing – review & editing. KN: Conceptualization, Funding acquisition, Supervision, Visualization, Writing – original draft.
