# Supplementary material for: The ST2+ Treg/amphiregulin axis protects from immune-mediated hepatitis
Source: Front Immunol. 2024 Mar 20;15:1351405. doi: 10.3389/fimmu.2024.1351405 (PMC10987816; doi:10.3389/fimmu.2024.1351405)
Supplement: Supplementary file 1 [file DataSheet_1.pdf]

Supplementary Material

The ST2^{+} Treg/amphiregulin axis protects from immune-mediated hepatitis

Selina Wachtendorf[1,2†], Fitriasari Jonin[1,2†], Aaron Ochel[1], Fabian Heinrich[3,4], Astrid M. Westendorf[5], Gisa Tiegs[1,2], Katrin Neumann[1,2]

[1]Institute of Experimental Immunology and Hepatology, University Medical Center Hamburg-Eppendorf, Hamburg, Germany

[2]Hamburg Center for Translational Immunology, University Medical Center Hamburg-Eppendorf, Hamburg, Germany

[3]Institute of Legal Medicine, University Medical Center Hamburg-Eppendorf, Hamburg, Germany

[4]Center for Data and Statistical Science for Health, London School of Hygiene and Tropical Medicine, London, UK

[5]Institute of Medical Microbiology, University Hospital Essen, University of Duisburg-Essen, Essen, Germany.

[†]These authors contributed equally to this work and share first authorship

Table of content: 7 Figures, 3 Tables

Table S1: Antibodies used for cell surface analysis by flow cytometry.

Target	Fluorochrome	Clone	Distributor
TCRβ	PE-Cy7, FITC	H57-597	BioLegend
CD4	BV 711	RM4-5	BioLegend
CD8	APC	53-6.7	BioLegend
ST2	PE-Cy7, PerCP-eF710	RMST2-2	ThermoFisher Scientific
CD25	BV 785, PE, PE-Cy7	PC61	BioLegend
KLRG1	BV 605	2F1/KLRG1	BioLegend
ICOS	FITC	7E.17G9	ThermoFisher Scientific
PD-L1	BV 421	10F.9G2	BioLegend
TIGIT	BV 421	1G9	BioLegend
CD103	BV 711	2E7	BioLegend
CD69	APC	H1.2F3	BioLegend
CCR7	PE	4B12	BioLegend

Table S2: Antibodies used for intracellular and intranuclear analysis by flow cytometry.

Target	Fluorochrome	Clone	Distributor
Foxp3	AF647<br>PerCP-Cy5.5	MF-14<br>FJK-16s	BioLegend<br>ThermoFisher Scientific
AREG	Biotin	polyclonal	R&D Systems
Streptavidin	BV 785	-	BioLegend
Ki-67	FITC	REA183	Miltenyi Biotec
CTLA-4	PE	UC10-4F10-11	BD Pharmingen
IFNγ	PE-CF594<br>BV 711	XMG1.2<br>XMG1.2	BD Horizon<br>BioLegend
TNFα	V450<br>PE-Cy7	MP6-XT22<br>MP6-XT22	BD Horizon<br>BioLegend

Table S3: Sequences of the primer used for mRNA analysis.

Target	Forward primer<br>Reverse primer	Amplicon length	Annealing temperature
Il33<br>(NM_001164724.1)	5'-ATGGGAAGAAGCTGATGGTG-3'<br>3'-CCGAGGACTTTTTGTGAAGG-5'	150	60°C
Areg<br>(NM_009704.4)	5'-GGTCTTAGGCTCAGGCCATTA-3'<br>3'-AGAGTTCACTGCCAGAAGGC-5'	161	60°C
Actb<br>(NM_007393)	5'-TATTGGCAACGAGCGGTTCC-3'<br>3'-GGCATAGAGGTCTTTACGGATGTC-5'	180	60°C

SUPPLEMENTARY FIGURE 1

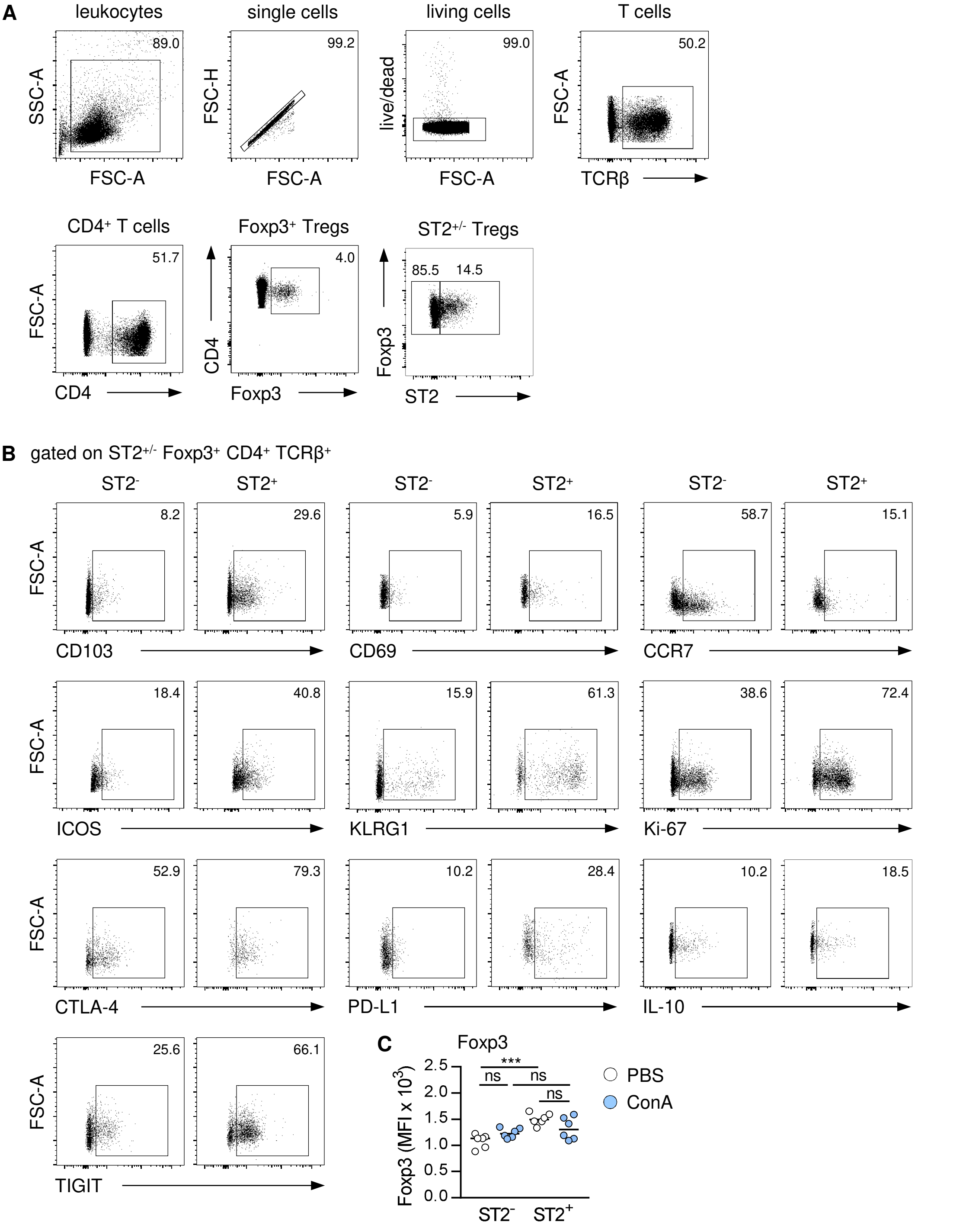

Supplementary Figure 1. Phenotype of hepatic $ST2^+$ $Foxp3^+$ Tregs. **(A)** Gating strategy of hepatic $ST2^+$ and $ST2^-$ $Foxp3^+$ Tregs. **(B)** Phenotype of $ST2^+$ and $ST2^-$ $Foxp3^+$ Tregs was analyzed in naïve WT mice. Representative dot plots and frequencies of at least two experiments are shown. (C) C57BL/6 mice were treated with ConA and analyzed one day later. Mean fluorescent intensity (MFI) of Foxp3 was determined in $ST2^+$ and $ST2^-$ $Foxp3^+$ Tregs. Medians of two individual experiments with 3 mice per group and experiment are shown. ***$p < 0.001$; ns: not significant.

SUPPLEMENTARY FIGURE 2

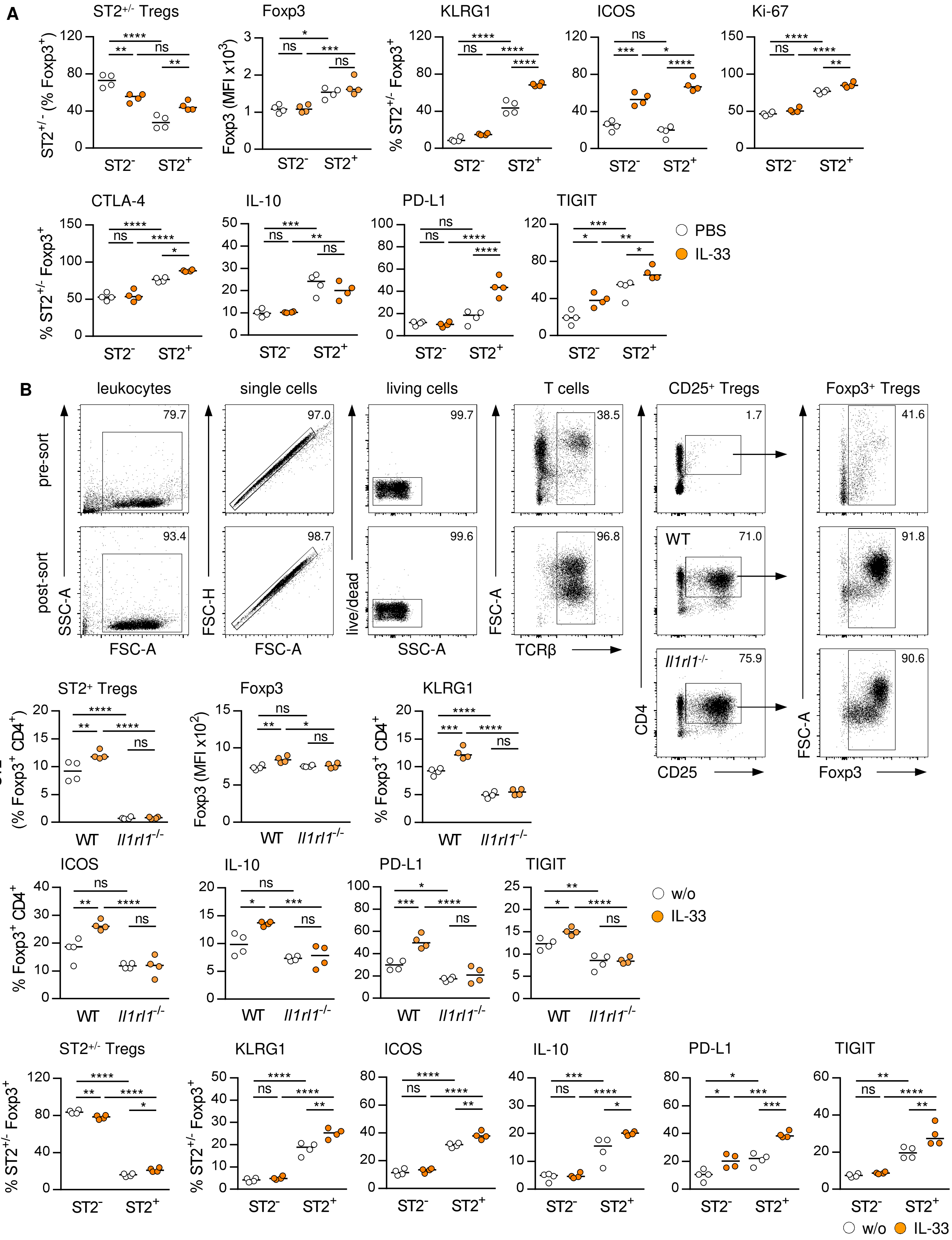

Supplementary Figure 2. IL-33 activates ST2+ Foxp3+ Tregs in vitro. **(A)** WT mice were treated with IL-33 on four consecutive days. Frequency and phenotype of hepatic ST2+ and ST2- Foxp3+ Tregs were analyzed. 4 mice per group and experiment are shown. **(B)** CD25+ CD4+ Tregs were isolated from spleen and lymph nodes of WT and *Il1rl1*-/- mice by MACS. Gating strategy and purity of isolated Tregs are depicted. Frequency of CD25+ CD4+ T cells that express Foxp3 is shown. **(C, D)** CD25+ Tregs from WT and *Il1rl1*-/- mice were cultured in presence IL-33 for 1.5 days. **(C)** Phenotype of Foxp3+ Tregs was analyzed. **(D)** Phenotype of ST2+ and ST2- Foxp3+ Tregs from WT mice was determined. Medians of one out of two individual experiments are shown. 4 well per sample were seeded. *p< 0.05; **p< 0.01; ***p< 0.001; ****p< 0.0001; ns: not significant; w/o: without; MFI: mean fluorescent intensity.

SUPPLEMENTARY FIGURE 3

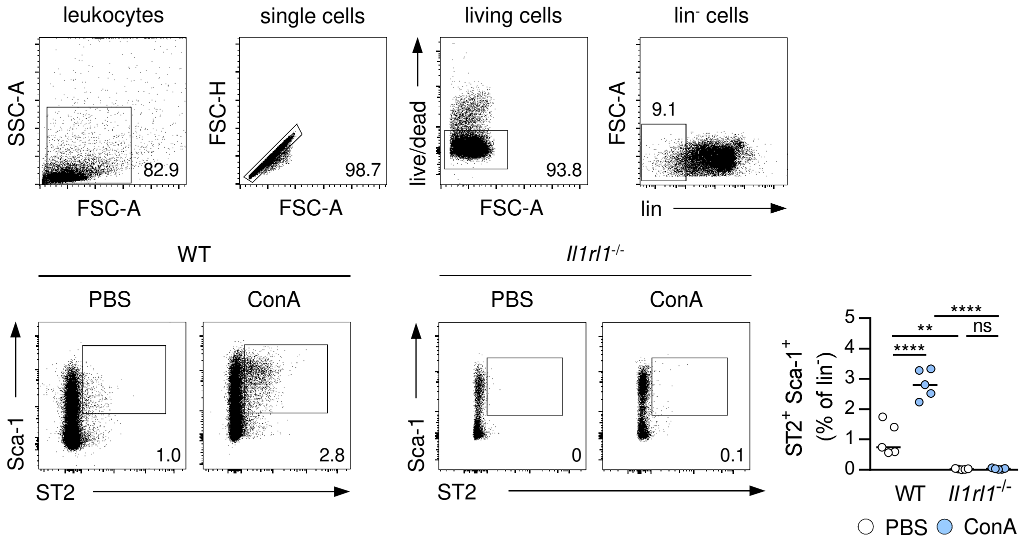

Supplementary Figure 3. IL-33 activates hepatic ILC2s in vivo. Immune-mediated hepatitis was induced in WT and *Il1rl1*$^{-/-}$ mice, which were analyzed one day later. Gating strategy and frequencies of $ST2^{+}$ Sca-1^{+} lin^{-} ILC2s from livers of WT and *Il1rl1*$^{-/-}$ mice. Medians of one out of two individual experiments with 5 mice per group and experiment are shown. *p< 0.05; **p< 0.01; ***p< 0.001; ****p< 0.0001; ns: not significant.

SUPPLEMENTARY FIGURE 4

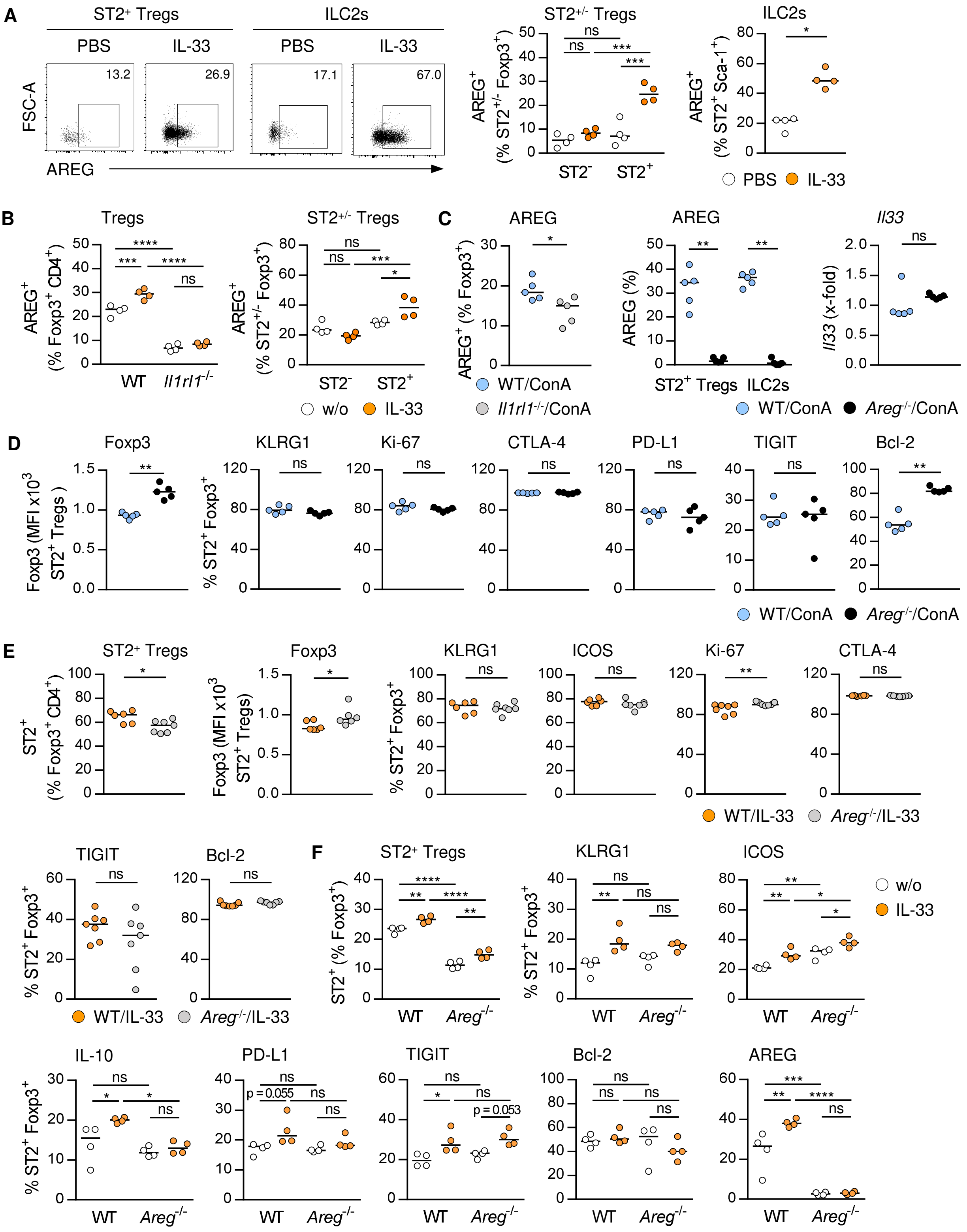

Supplementary Figure 4. Phenotype of ST2+ Tregs from *Areg*-/- mice. **(A)** WT mice were treated with IL-33 on four consecutive days. Expression of AREG was analyzed in hepatic ST2+ and ST2- Tregs and ILC2s. Representative dot plots are shown. **(B)** CD25+ CD4+ Tregs were isolated from WT and *Il1rl1*-/- mice and cultured in presence of IL-33 for 1.5 days. Expression of AREG was analyzed. **(C)** Immune-mediated hepatitis was induced in WT, *Il1rl1*-/- and *Areg*-/- mice, which were analyzed 24 hours later. AREG expression was determined in hepatic Foxp3+ Tregs, ST2+ Tregs and ILC2s. Hepatic *Il33* mRNA was analyzed in *Areg*-/- and WT mice. **(D)** Phenotype of hepatic ST2+ Tregs was analyzed. **(E)** WT and *Areg*-/- mice were treated with IL-33 on four consecutive days. Phenotype of hepatic ST2+ Tregs was analyzed. **(F)** CD25+ CD4+ Tregs were isolated from WT and *Areg*-/- mice and cultured in presence of IL-33 for 1.5 days. Phenotype of ST2+ Tregs was analyzed. Medians of one out of two individual experiments 5-7 mice per group and experiment are shown. *p< 0.05; **p< 0.01; ***p< 0.001; ****p< 0.0001; ns: not significant; w/o: without; MFI: mean fluorescent intensity.

SUPPLEMENTARY FIGURE 5

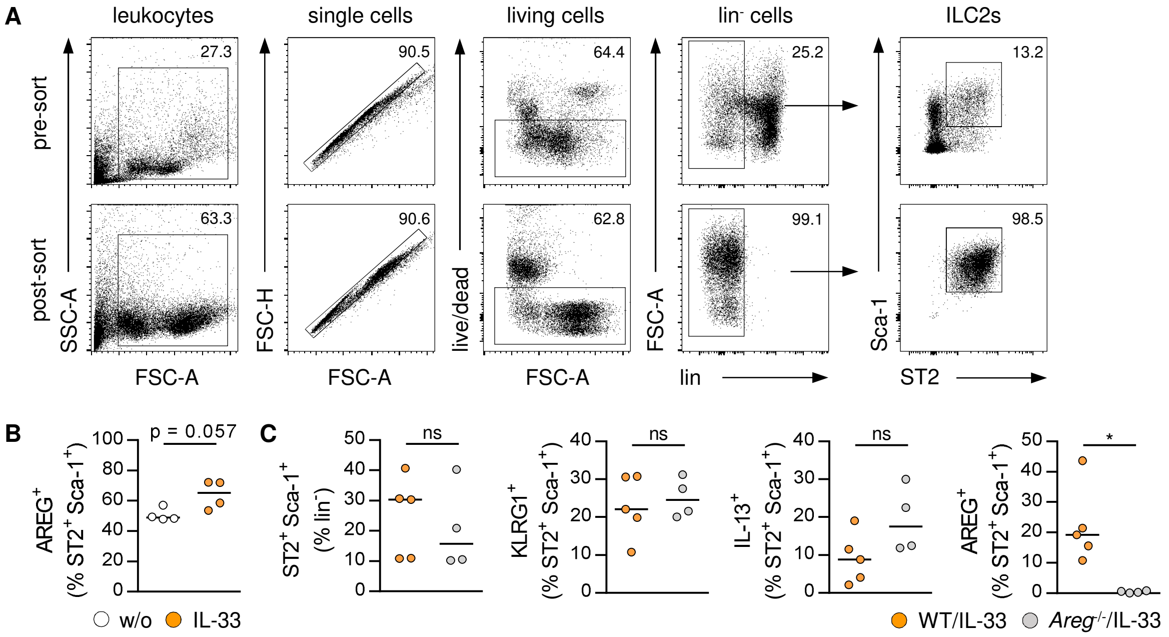

Supplementary Figure 5. IL-33-induced phenotype of hepatic ILC2s in *Areg*$^{-/-}$ mice. **(A)** WT mice were treated with IL-33 on four consecutive days. Hepatic lineage-negative (lin^{-}) cells were enriched by MACS. ST2^{+} Sca-1^{+} lin^{-} ILC2s were isolated by FACS. Gating strategy and purity of isolated hepatic ILC2s are shown. **(B)** Hepatic ILC2s isolated from WT mice were cultured in presence of IL-33 for 3.5 days. Expression of AREG was determined. (C) WT and *Areg*$^{-/-}$ mice were treated with IL-33 on four consecutive days. Frequency and phenotype of hepatic ILC2s were analyzed. Medians of one out of two independent experiments with 4-5 mice per group and experiment are shown. In vitro, 4 wells per sample were seeded. *$p < 0.05$; ns: not significant.

SUPPLEMENTARY FIGURE 6

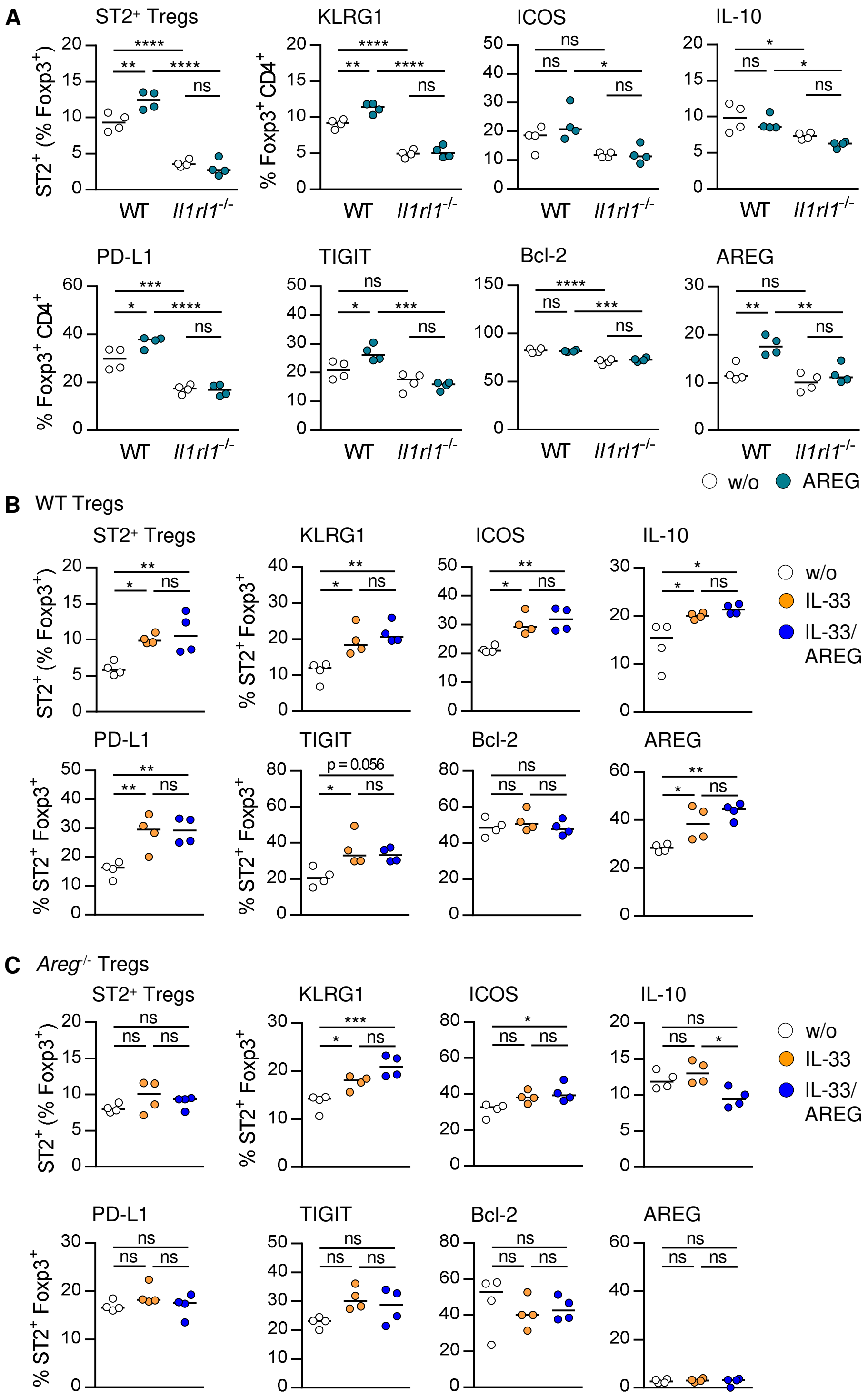

Supplementary Figure 6. AREG-induced phenotype of ST2+ Foxp3+ Tregs. **(A)** CD25+ CD4+ Tregs were isolated from WT and *Il1rl1-/-* mice and cultured in presence of AREG for 1.5 days. Phenotype of Foxp3+ Tregs was analyzed. **(B, C)** CD25+ CD4+ Tregs were isolated from **(B)** WT and **(C)** *Areg-/-* mice and cultured in presence of IL-33 or IL-33/AREG. Phenotype of ST2+ Foxp3+ Tregs was determined. Medians of one out of two independent experiments are shown. 4 wells per sample were seeded. *p< 0.05; **p< 0.01; ***p< 0.001; ****p< 0.0001; ns: not significant; w/o: without.

SUPPLEMENTARY FIGURE 7

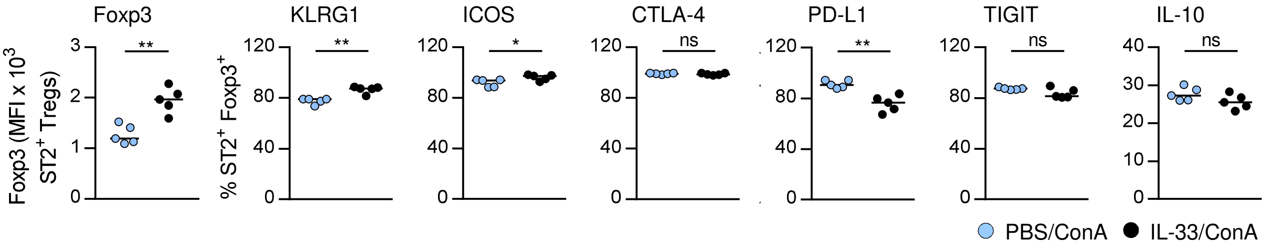

Supplementary Figure 7. Phenotype of $ST2^+$ Tregs in immune-mediated hepatitis after IL-33 pre-treatment. WT mice were treated with IL-33 on three consecutive days. On the fourth day, mice received ConA and were analyzed 24 hours later. Phenotype of hepatic $ST2^+$ $Foxp3^+$ Tregs was analyzed. Medians of one out of two independent experiments with 5 mice per group and experiment are shown. *p< 0.05; **p< 0.01; ns: not significant; MFI: mean fluorescent intensity.
